# Accu16S/AccuITS: Accurate and broadly applicable amplicon sequencing for absolute microbiome quantification

**DOI:** 10.1002/imt2.70116

**Published:** 2026-03-02

**Authors:** Defeng Bai, Ou Fang, Caihua Li, Bin Cai, Xingyan Tan, Mengmeng Jiang, Bin Gan, Jinxia Fu, Yunyun Gao, Ying Wang, Yong‐Xin Liu

**Affiliations:** ^1^ State Key Laboratory of Tropical Crop Breeding, Genome Analysis Laboratory of the Ministry of Agriculture and Rural Affairs, Agricultural Genomics Institute at Shenzhen, Chinese Academy of Agricultural Sciences Shenzhen China; ^2^ Shanghai GeneCowin Biotechnology Co., Ltd. Shanghai China; ^3^ School of Ecology and Nature Conservation, Beijing Forestry University Beijing China; ^4^ Institute of Genomics and Precision Medicine, School of Medical Technology, Gannan Medical University Ganzhou China

## Abstract

Traditional 16S rRNA gene and Internal Transcribed Spacer region amplicon sequencing provides only relative abundance, often leading to biased ecological interpretations. To overcome this limitation, we developed Accu16S/AccuITS, an absolute quantification method for bacterial and fungal amplicons based on synthetic internal spike‐in DNA with known copy numbers. By adding internal standards prior to Polymerase Chain Reaction and sequencing, absolute microbial abundances can be calculated using standard curve regression. Accu16S/AccuITS exhibits sensitivity and consistency comparable to quantitative Polymerase Chain Reaction and is applicable to diverse sample types. A single sequencing run simultaneously yields relative abundance, total absolute abundance, and taxon‐specific absolute abundance. Case studies across diverse ecosystems demonstrate that absolute quantification provides ecologically and functionally meaningful insights beyond those obtained from relative abundance analyses.

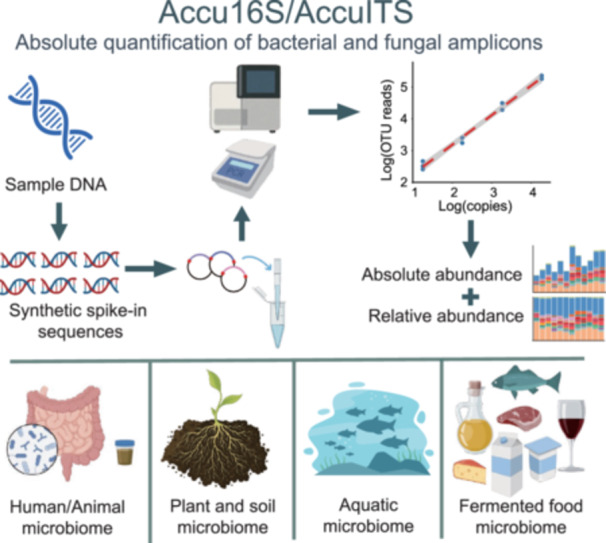

## AUTHOR CONTRIBUTIONS

**Defeng Bai**: Writing—original draft; methodology; validation; visualization; writing—review and editing; software; formal analysis; investigation. **Ou Fang**: Investigation; writing—original draft; methodology; validation; visualization; writing—review and editing; software; formal analysis. **Caihua Li**: Investigation; writing—original draft; methodology; validation; visualization; writing—review and editing; software; formal analysis. **Bin Cai**: Validation; methodology; writing—review and editing; software. **Xingyan Tan**: Methodology; validation; writing—review and editing; software. **Mengmeng Jiang**: Methodology; validation; writing—review and editing; software. **Bin Gan**: Methodology; validation; writing—review and editing; software. **Jinxia Fu**: Methodology; validation; writing—review and editing; software. **Yunyun Gao**: Methodology; writing—review and editing; software; validation; visualization. **Ying Wang**: Methodology; validation; writing—review and editing; software; conceptualization; funding acquisition; project administration; resources; supervision. **Yong‐Xin Liu**: Conceptualization; funding acquisition; methodology; validation; writing—review and editing; software; project administration; resources; supervision. All authors have read the final manuscript and approve it for publication.

## CONFLICT OF INTEREST STATEMENT

The work related to this research has obtained a national patent certificate in China. The patent number is ZL 2019 1 0295389.X. The patentee is Shanghai GeneCowin Biotechnology Co., Ltd.

## ETHICS STATEMENT

No animals or humans were involved in this study.

To the Editor,

Microorganisms are ubiquitous and play essential roles in host physiology and ecosystem function across humans [1], animals [2, 3], plants [4], and diverse environments [5]. Amplicon sequencing of marker genes such as 16S rRNA gene (bacteria) and Internal Transcribed Spacer (ITS; fungi) region has become a cornerstone for profiling microbial communities [6, 7]. However, conventional short‐read sequencing offers limited species‐level resolution and yields only relative abundance, which can misrepresent microbial dynamics [8]. Although third‐generation full‐length sequencing technologies (e.g., Nanopore or Pacific Biosciences (PacBio)) enhance taxonomic resolution, they still fall short because they lack absolute abundance information [9]. Previous studies have noted that host DNA contamination can bias relative abundance‐based quantification [4]. While host DNA contamination primarily reduces the proportion of effective sequencing reads, thereby compromising coverage and taxonomic resolution, it does not inherently result in a loss of absolute abundance information. Instead, the fundamental limitation of relative abundance data lies in its compositional nature, where the closed‐sum constraint can induce spurious correlations and misleading ecological interpretations [8]. Notably, in human gut microbiome studies, absolute abundance has been shown to correlate more closely with host traits than relative abundance‐based measures [10].

To overcome these challenges, absolute quantification methods are increasingly used to provide more accurate absolute abundance measurements [11]. While quantitative Polymerase Chain Reaction (qPCR) and flow cytometry have been applied, they suffer from primer biases and background interference, respectively [10]. Internal synthetic spike‐in strategies have emerged as a more robust solution, offering greater sensitivity and consistency across sample types [12, 13, 14]. In this study, we developed an internal spike‐in‐based absolute quantification method for bacterial and fungal amplicons. By co‐amplifying spike‐ins with known copy numbers alongside sample DNA, we constructed standard curves to estimate operational taxonomic unit (OTU) or amplicon sequence variant (ASV)‐level absolute abundances without relying on external references, leveraging the high throughput and scalability of next‐generation sequencing.

## AN OVERVIEW OF ACCU16S/ACCUITS WORKFLOW, PRINCIPLE, AND ADVANTAGES

**Figure 1:**
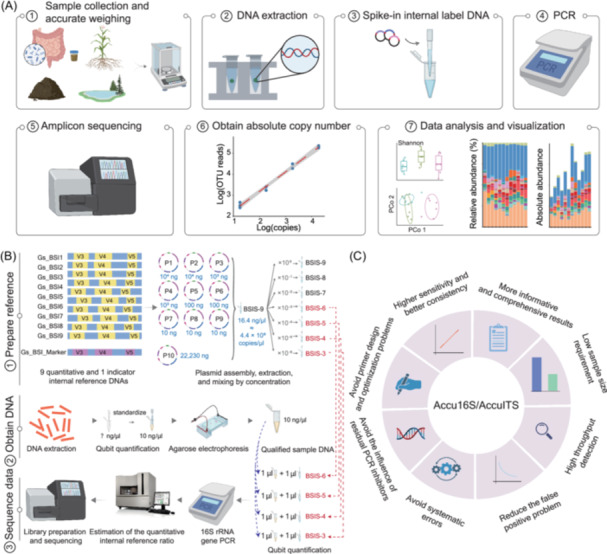
Overview and advantages of the Accu16S/AccuITS method for absolute microbial quantification. (A) Workflow of Accu16S/AccuITS: synthetic spike‐in sequences with known copy numbers are added after DNA extraction, co‐amplified with microbial DNA, and used to construct a standard curve for absolute quantification. (B) Principle of Accu16S/AccuITS for profiling microbial community composition and estimating absolute abundance (16S rRNA gene amplicon sequencing shown as an example). (C) Key advantages of Accu16S/AccuITS, including high accuracy, scalability, and broad applicability. Graphics created with BioRender (https://app.biorender.com/).

The Accu16S/AccuITS pipeline is an optimized absolute quantification workflow built upon standard microbiome analysis, supporting a wide range of sample types, including medical (e.g., feces, tissues, swabs), food (e.g., liquor Daqu, fermented fish), environmental (e.g., marine plastics, sediments), agricultural (e.g., rhizosphere soils), and ecological samples (e.g., altitude or climate‐related soil microbiomes) (Figure 1A). After DNA extraction, spike‐in sequences with known copy number are added, and both microbial and spike‐in DNA are co‐amplified for 16S rRNA gene or ITS library preparation and sequencing on Illumina or Beijing Genomic Institute platforms (Figure 1B). Absolute OTU/ASV abundances are calculated via standard curves derived from spike‐in reads. To facilitate downstream analysis, we provide shell scripts and a pipeline freely available on GitHub (Accu16S_ITS directory in https://github.com/YongxinLiu/EasyAmplicon) and an online analysis platform (http://cloud2.genecowin.com).

To validate the accuracy of Accu16S/AccuITS, we compared the spike‐in‐based approach with qPCR and Applied Biosystems (ABI) 3730 using ZymoBIOMICS microbial community (ZYMO) standards. Accu16S showed a strong correlation with qPCR (*R* ^2^ = 0.922; Figure S1A) and demonstrated superior stability in the presence of humic acid inhibitors compared with qPCR (Figure S1B). In addition, absolute quantification results obtained by Accu16S were consistent with theoretical values, qPCR, and ABI 3730 measurements across three standards (*p* > 0.05; Figure S2; Table S1), confirming the accuracy and robustness of the method. A key innovation of Accu16S/AccuITS is the use of the ABI 3730 genetic analyzer to measure PCR amplicons by capillary electrophoresis, where peak areas reflect molar quantities and allow estimation of spike‐in proportions based on target‐to‐indicator peak ratios (Figure S3). The spike‐in proportions inferred from peak areas accurately matched those in the final sequencing libraries (Table S2), and analysis of 24 soil samples demonstrated highly reproducible 16S rRNA gene copy number quantification across replicates (Figure S4 and Table S3). Additional methodological details and the data tables required for calculations are provided in the Supplementary Materials (Tables S4–S6).

Accu16S/AccuITS is a widely adopted method for the absolute quantification of microbial amplicon sequencing data and offers several advantages. First, a single sequencing run can simultaneously generate three types of data (relative abundance, total, and absolute abundance at each taxonomic level). Second, it requires only a small sample amount, minimizing the risk of sequencing failure due to limited material. Third, it provides high detection throughput for diverse microbial taxa. Fourth, it enables cross‐validation between relative and absolute data, thereby reducing false positives. Fifth, it avoids platform‐specific biases commonly observed in qPCR. Sixth, it mitigates the effects of PCR inhibitors introduced during DNA extraction. Seventh, it eliminates the challenges associated with primer design and optimization that are typical of qPCR experiments. Finally, it achieves consistency through internal labeling compared to qPCR (Figure 1C).

## APPLICATIONS OF ACCU16S/ACCUITS IN GUT, ENVIRONMENT, OR FOOD MICROBIOTA

The Accu16S method for bacterial amplicons was developed in 2018, followed by AccuITS for fungal amplicons in 2021. In June 2023, the combined Accu16S/AccuITS approach was granted a national invention patent in China (Patent number: ZL 2019 1 0295389.X). It has since been used in hundreds of studies, contributing to many innovative findings in microbiome research (https://github.com/YongxinLiu/EasyAmplicon/blob/master/Accu16S_ITS/Representative_papers.xlsx). By adding the internal standard directly to the extracted DNA, our approach minimizes potential biases arising from differences in sample matrices or microbial adaptability across distinct sample types. This study shows five previously published examples highlighting its broad application of absolute abundance quantification.

**Figure 2:**
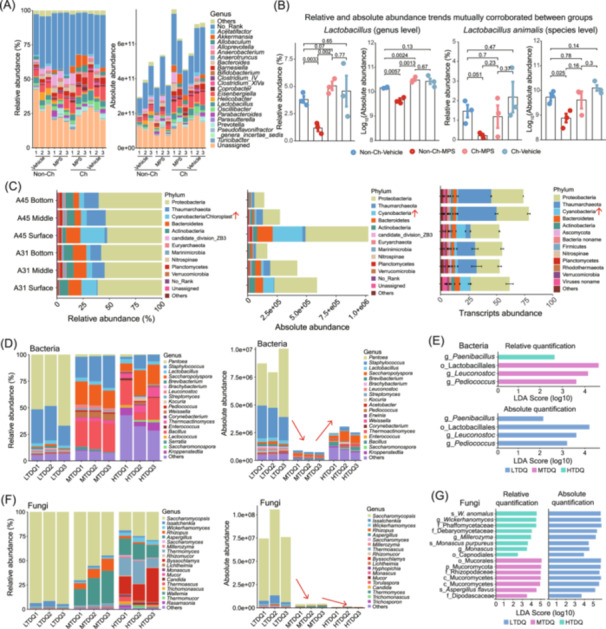
Case studies illustrating Accu16S/AccuITS applications across diverse microbiome contexts. (A, B) In a glucocorticoid‐induced osteonecrosis of the femoral head (ONFH) model, Accu16S identified key taxa, with absolute and relative abundances showing consistent trends across groups, supporting result reliability (Non‐Ch and Ch represent non‐cohoused and cohoused mice, respectively, while MPS and Vehicle denote treatment with methylprednisolone and a vehicle solution without active ingredients, respectively). Student's *t* test (unpaired, two‐tailed) was used for analyzing the differences between two groups. (C) In seawater samples, absolute quantification closely matched total prokaryotic counts measured by flow cytometry and outperformed relative and transcript‐based approaches. (D, E) In *Daqu* fermentation, Accu16S‐based absolute and relative abundance analyses produced distinct bacterial Linear Discriminant Analysis Effect Size (LEfSe) results, demonstrating the added value of absolute quantification (LTDQ, MTDQ, and HTDQ indicate low‐, medium‐, and high‐temperature *Daqu*). (F, G) In *Daqu* fermentation, AccuITS revealed pronounced differences between relative and absolute abundance in fungal LEfSe analyses. The red arrows indicate the trend of absolute abundance across different groups.

A study on gut microbiota used Accu16S absolute quantification to explore the gut microbiota role in glucocorticoid‐induced osteonecrosis [15]. Relative and absolute fecal microbiota abundances at the genus level were compared in cohoused (Ch) and non‐cohoused (Non‐Ch) mice treated with vehicle (solution without active ingredients) or methylprednisolone (MPS) (Figure 2A and Table S7). Results showed consistent trends between relative and absolute abundances, especially for *Lactobacillus* (genus level) and *Lactobacillus animalis* (species level) across groups (Figure 2B). A specific example is the relative and absolute abundances of *Lactobacillus animalis* and *Lactobacillus* showed consistent trends across treatment groups, with significantly lower levels in Non‐Ch‐MPS versus control, validating the reliability of combining relative and absolute quantification.

To study microbial carbon and nitrogen cycling in seawater, researchers compared relative, absolute, and transcript abundances across water depths [16]. Relative data showed nine dominant phyla, with Cyanobacteria enriched at the surface and Thaumarchaeota in the middle waters. Absolute quantification revealed Proteobacteria, Cyanobacteria, and Thaumarchaeota made up 77.2% of total abundance, with Proteobacteria and Cyanobacteria declining with depth. Absolute quantification and metatranscriptome analysis showed consistent results that Cyanobacteria decreased with deeper water, underscoring the ecological value of absolute quantification (Figure 2C and Table S8).

In the context of fermented food microbiota, researchers applied the Accu16S/AccuITS to systematically assess the bacterial and fungal communities in *Daqu* and their roles in the fermentation process [17]. A comparative analysis revealed substantial discrepancies between relative and absolute abundance results of bacterial and fungal communities (Figure 2D,F and Table S9), particularly in Linear Discriminant Analysis Effect Size (LEfSe) analyses across groups. LEfSe analysis identified bacterial and fungal taxa differing significantly among LTDQ (low‐temperature), MTDQ (medium‐temperature), and HTDQ (high‐temperature) *Daqu* groups. While linear discriminant analysis (LDA) scores were similar between relative and absolute abundance, the enrichment patterns of these taxa differed between relative and absolute quantification methods (Figure 2E,G and Table S6). Absolute quantification offers essential insights when total microbial biomass varies and reduces biases inherent in relative measurements, highlighting its importance for accurate interpretation of microbiome data.

In addition, we also provided examples for the application of Accu16S/AccuITS to uncover plant‐microbe interactions, including microbial recruitment in continuous cropping potatoes (Table S10), and nutrient‐cycling bacteria in sand fixation (Figure S5 and Table S11).

Accu16S/AccuITS provides an accurate, scalable, and widely applicable solution for absolute microbial abundance quantification. Some spike‐in methods are limited to specific sample types (e.g., root [12], rhizosphere [13], gut [18]). Non‐spike‐in approaches such as qPCR [10] have low throughput. Accu16S/AccuITS delivers comprehensive data (count, relative, absolute abundance) in a single run. It supports high‐throughput analysis with low input requirements and maintains high taxonomic resolution across diverse sample types. In addition, referring to our previously published pipelines [7, 19, 20], we also provide shell scripts that include sequence data processing, host removal, sequence quantification, standard curve construction, absolute quantification and correction, and visualization options. By offering both an online platform and flexible shell scripts, Accu16S/AccuITS accommodates diverse user needs and offers greater flexibility.

Although the Accu16S/AccuITS method demonstrates broad applicability and achieves accuracy comparable to qPCR, it also has several limitations. First, the addition of spike‐in sequences to each sample increases procedural complexity and cost. Second, variations in DNA extraction efficiency among samples may influence quantification accuracy. Third, the limit of detection may be restricted when analyzing very low‐biomass samples. Finally, we only compared the accuracy of qPCR, capillary electrophoresis and Accu16S in amplicon absolute quantification, we will further compare Accu16S/AccuITS with other published spike‐in‐based absolute quantification methods [12, 13] in the future to optimize amplicon absolute quantification methods. Despite the limitations mentioned above, the main advantage of Accu16S/AccuITS lies in its high‐throughput, versatility (it can be applied to different research directions) and its suitability for large‐scale sample analysis. Accu16S/AccuITS provides a user‐friendly option for amplicon absolute quantification.

Looking forward, we aim to expand Accu16S/AccuITS by integrating third‐generation full‐length amplicon and metagenomic sequencing to improve species‐level resolution in absolute quantification. The spike‐in strategy itself could be further optimized by developing universal spike‐in standards that cover multiple marker genes (e.g., 16S rRNA gene, ITS, functional genes) to achieve cross‐kingdom quantification. Moreover, integrating spike‐in‐based absolute quantification with automated library preparation and digital PCR validation could enable high‐throughput, standardized, and more reproducible absolute microbiome profiling in the future. Further optimization, such as integrating Unique Molecular Identifiers (UMIs), could reduce PCR amplification bias by labeling original DNA molecules and enabling correction of PCR duplicates. Although UMIs increase library complexity, cost, and data‐processing demands, their incorporation may further improve quantitative accuracy beyond the current spike‐in‐based calibration used in Accu16S/AccuITS. We will also enhance our cloud platform by adding new modules for data analysis and visualization, and enrich the shell script with faster, more flexible tools for generating high‐quality, publication‐ready figures. These efforts will further streamline the absolute quantification workflow and support broader applications in microbiome research.

## Supporting information


**Figure S1:** Comparison between Accu16S/AccuITS and traditional Polymerase Chain Reaction (qPCR).
**Figure S2:** Comparison of Accu16S method with other methods for absolute quantification of microorganisms.
**Figure S3:** The capillary electrophoresis results using PeakScan software.
**Figure S4:** The absolute copy number of 16S rRNA gene amplicon microorganisms at the level in real soil samples.
**Figure S5:** Additional representative studies using Accu16S for microbial absolute quantification.

**Table S1:** Standard curves for microbial absolute abundance quantification using Accu16S were constructed using ZymoBIOMICS microbial community (ZYMO) gut samples.
**Table S2:** Estimated vs. actual value of the proportion of “quantitative internal references”.
**Table S3:** Standard curves for microbial absolute abundance quantification using Accu16S were generated based on 24 soil samples.
**Table S4:** Formulation of internal reference sequence mixture.
**Table S5:** Copy number of each quantitative reference sequence in Bacterial Spike‐In Standards (BSIS) at 10‐fold serial dilution.
**Table S6:** Accu16S/AccuITS compatible 16S rRNA gene amplification regions and corresponding primers.
**Table S7:** Standard curves for calculating microbial absolute abundance using Accu16S were generated using samples from Chen et al. (2022).
**Table S8:** Standard curves for calculating microbial absolute abundance using Accu16S were generated using samples from Han et al. (2022).
**Table S9:** Standard curves for calculating microbial absolute abundance using Accu16S/AccuITS were generated using samples from Kang et al. (2022).
**Table S10:** Standard curves for calculating microbial absolute abundance using Accu16S were generated using samples from Ma et al. (2025).
**Table S11:** Standard curves for calculating microbial absolute abundance using Accu16S were generated using samples from Liu et al. (2022).

## Data Availability

The data used in this study are available in the Accu16S_ITS directory of GitHub https://github.com/YongxinLiu/EasyAmplicon. Supplementary materials (methods, figures, tables, graphical abstract, slides, videos, Chinese translated version, and update materials) may be found in the online DOI or iMeta Science http://www.imeta.science/.
