# Supplementary material for: Accu16S/AccuITS: Accurate and broadly applicable amplicon sequencing for absolute microbiome quantification
Source: Imeta. 2026 Mar 2;5(2):e70116. doi: 10.1002/imt2.70116 (PMC13147958; doi:10.1002/imt2.70116)
Supplement: Supplementary file 1 — Figure S1: Comparison between Accu16S/AccuITS and traditional Polymerase Chain Reaction (qPCR). Figure S2: Comparison of Accu16S method with other methods for absolute quantification of microorganisms. Figure S3: The capillary electrophoresis results using PeakScan software. Figure S4: The absolute copy number of 16S rRNA gene amplicon microorganisms at the level in real soil samples. Figure S5: Additional representative studies using Accu16S for microbial absolute quantification. [file IMT2-5-e70116-s001.docx]

Supporting information to

**Accu16S/AccuITS: Accurate and broadly applicable amplicon sequencing for absolute microbiome quantification**

**Running title**: Accu16S/AccuITS for absolute microbial quantification

Defeng Bai^1#^, Ou Fang^2#^, Caihua Li^2#^, Bin Cai^2^, Xingyan Tan^2^, Mengmeng Jiang^2^, Bin Gan^2^, Jinxia Fu^2^, Yunyun Gao^3^, Ying Wang^2*^, Yong-Xin Liu^1, 4*^

1. State Key Laboratory of Tropical Crop Breeding, Genome Analysis Laboratory of the Ministry of Agriculture and Rural Affairs, Agricultural Genomics Institute at Shenzhen, Chinese Academy of Agricultural Sciences, Shenzhen 518120, China
2. Shanghai GeneCowin Biotechnology Co., Ltd., Shanghai 201401, China
3. School of Ecology and Nature Conservation, Beijing Forestry University, Beijing 100083, China
4. Institute of Genomics and Precision Medicine, School of Medical Technology, Gannan Medical University, Ganzhou 341000‌, China

^#^These authors contributed equally: Defeng Bai, Ou Fang, Caihua Li

^*^Correspondence: [57571315@qq.com](mailto:57571315@qq.com) (Ying Wang), [liuyongxin@caas.cn](mailto:liuyongxin@caas.cn) (Yong-Xin Liu)

**METHODS**

The Accu16S/AccuITS method integrates experimental, bioinformatic, and visualization steps to quantify microbial absolute abundance. By introducing internal spike-in DNA with known copy numbers after extraction and co-amplifying it with microbial DNA, a standard curve is generated to calculate absolute operational taxonomic unit (OTU) or amplicon sequence variant (ASV) copy numbers, which are further adjusted by DNA input, library concentration, and sample weight. We provide a complete shell script covering raw data processing to feature table generation, along with five case studies (animal gut, plant rhizosphere, seawater, fermented food, and soil microbiomes) demonstrating the method’s application and interpretation.

To evaluate the performance and reproducibility of the method, 24 soil samples from eight sites were subjected to absolute quantification of bacterial 16S rRNA genes using Accu16S. After DNA extraction and normalization, samples were spiked with Bacterial Spike-In Standards (BSIS) sequences, amplified, and sequenced, and total 16S rRNA gene copy numbers per gram of soil were calculated based on a spike-in standard curve. The accuracy of Accu16S was further validated by comparison with quantitative Polymerase Chain Reaction (qPCR) and Applied Biosystems (ABI) 3730 capillary electrophoresis using three ZymoBIOMICS Microbial Community (ZYMO) standards. The data analysis and visualization codes provided by MicrobiomeStatPlots are used for plotting and result interpretations [1]. Reproducible code and visual outputs are included to guide users in diverse research fields.

**Spike-in design for amplicon absolute quantification**

This method employs an artificially synthesized internal standard sequence mixture for absolute quantification of 16S rRNA gene copy numbers. The synthesized quantitative reference and indicator internal standard DNAs can be in the form of plasmids, chemically synthesized DNA fragments, or Polymerase Chain Reaction (PCR) products. The quantitative reference sequences are used to construct standard curves for calculating the absolute copy number of 16S rRNA genes across taxonomic units. Each reference sequence is designed with three conserved motifs, C2 (CCAGACTCCTACGGG(A/T/C/G)GGC(A/T)GCAG), C3 (GTGCCAGC(A/C)GCCGCGGTAA(T/C)ACG), and C5 (GGATTAGA(A/T)ACCC(T/G/C)(A/T/G)GTAGTCC), arranged sequentially from the 5' to 3' end, separated by random sequences of approximately 157 ± 3 bp, 247 ± 3 bp, and 100 ± 3 bp in length, each with a GC content between 35% and 55%.

The indicator internal standard is used to evaluate the ratio between the quantitative reference sequences and the 16S rRNA gene copy number in samples prior to high-throughput sequencing. It consists of a single artificially synthesized DNA sequence with the following structure: from the 5' to 3' end, it contains the conserved motifs C2 (CCAGACTCCTACGGG(A/T/C/G)GGC(A/T)GCAG), C3 (GTGCCAGC(A/C)GCCGCGGTAA(T/C)ACG), C4 (GGATTAGA(A/T)ACCC(T/G/C)(A/T/G)GTAGTCC), and C5 (AAACT(T/C)AAA(T/G)GAATTGACGG), arranged sequentially. These motifs are separated by random sequences approximately 257 ± 50 bp, 347 ± 50 bp, and 200 ± 50 bp in length, each with a GC content between 35% and 55%.

The **Accu16S/AccuITS** quantitative standard includes nine specifically designed quantitative reference sequences that enable accurate detection of bacterial community composition and abundance. By mixing these reference sequences at tenfold serial ratios, the method allows simultaneous acquisition of both microbial compositional and absolute quantitative information from a single sequencing library. Additionally, one **indicator internal standard** was introduced to estimate the proportion of quantitative reference sequences before sequencing. Using these artificially synthesized sequences, we developed a capillary electrophoresis-based approach that estimates the ratio of quantitative reference sequences according to the relative peak areas of fluorescence signals, effectively overcoming limitations in previous methods caused by the inability to precisely control spike-in ratios. In brief, the nine quantitative spike-ins were designed to span a wide dynamic range of copy numbers, enabling accurate quantification across samples with varying absolute abundance, while the indicator sequence was used to monitor potential contamination and amplification efficiency. All sequences were generated de novo and screened by BLAST searches against public databases (NCBI nt/nr) to ensure no significant homology with known microbial genomes, thereby minimizing amplification bias and preventing misclassification during bioinformatic analysis.

The implementation of **Accu16S/AccuITS** involves several key steps. First, the **internal standard sequence mixture** (BSIS) is synthesized and prepared. Ten DNA sequences are chemically synthesized, including nine **quantitative reference sequences** (Gs_BSI1 to Gs_BSI9) and one **indicator internal standard** (Gs_BSI_Marker), which are then cloned into the **pUC19 plasmid vector**. Although plasmids were used as carriers in this study, the internal standards can also be provided in other forms, such as chemically synthesized DNA fragments or PCR products. Next, the plasmids are transformed into *Escherichia coli* **competent cells**, and single colonies are selected and cultured in Luria-Bertani (LB) medium for amplification. Plasmids are then extracted from the bacterial cultures, and their concentrations are accurately measured using a **Qubit fluorometer**. The nine quantitative reference plasmids are mixed according to the ratios listed in Table S7 and brought to a final volume of 2700 μL in tris(hydroxymethyl)aminomethane ethylenediaminetetraacetic acid (**TE) buffer** (10 mM Tris-Cl, 1 mM EDTA, pH 8.0), denoted as BSIS-9. The concentration of BSIS-9 is measured independently in three replicates, with an expected concentration of approximately 16.4 ng/μL, corresponding to a total copy number of ~4.4 × 10^9^ copies/μL (unit for liquid objects); if the measured concentration differs by more than 10%, the mixture must be re-prepared. Finally, BSIS-9 is serially diluted in tenfold steps, with sequence identifiers and corresponding copy numbers provided in Table S8. Among these, BSIS-7 to BSIS-4 are sufficient to meet the requirements of most experimental applications.

Next, the **BSIS mixture** is added to the samples and the proportion of BSIS is estimated. First, total DNA is extracted from each sample using any suitable method, ensuring that comparable amounts of sample are used and accurately recorded (*e.g.*, grams of soil, grams of feces, liters of wastewater), denoted as I (represent sample amounts used), along with the total DNA yield N (represent total DNA in ng). Second, DNA concentrations are accurately measured using a **Qubit fluorometer**, and each sample is normalized to 200 ng in 20 μL of **1× TE buffer**. Third, 2 μL of the normalized DNA is checked by gel electrophoresis to assess consistency among samples and evaluate DNA integrity. Samples with obvious concentration deviations are re-normalized, while degraded samples are considered quality control failures and excluded from further experiments. Fourth, 1 μL each of **BSIS-6, BSIS-5, BSIS-4, and BSIS-3** is added to 1 μL of normalized DNA from each sample, and PCR amplification is performed for 25 cycles using the primers listed in Table S9. Fifth, the PCR products are analyzed by gel electrophoresis to verify the presence of large indicator bands. Sixth, 1 μL of the tenfold-diluted PCR products is mixed with 9 μL **Hi-Di formamide** and 0.1 μL **LIZ500**, and analyzed on an **ABI 3730 sequencer** using the Short Tandem Repeat (STR) program. Seventh, the **PeakScan software (ABI)** is used to calculate the ratio of the peak area of the indicator band (HA) to that of the target band (HT) (HA/HT), which provides an estimate of the proportion of the quantitative reference sequences in the sample.

Next, **Index PCR and high-throughput sequencing** are performed. First, samples with an optimal proportion of quantitative reference sequences (10–60%) are selected for **Index PCR**, in which index sequences are added to both ends of the amplicon library to distinguish individual samples, along with the universal sequences required for **Illumina sequencing**. Second, the libraries are sequenced on the **Illumina HiSeq platform** with a read length of 2 × 250 bp, targeting approximately 10 million reads per sample.

Finally, the bacterial community composition and both relative and absolute abundances are obtained based on the **BSIS standard curve**. First, sequencing data are analyzed using conventional methods: sequences are clustered into OTUs at 97% and 99% identity thresholds, followed by taxonomic annotation through alignment against reference databases (*e.g.*, 16S rRNA gene databases or NCBI nt), providing the taxonomic composition from phylum to genus and the relative abundance of each taxon. Second, OTU representative sequences are aligned to the nine **quantitative reference sequences** to determine their read counts. Third, a standard curve is constructed with the read counts of the quantitative reference sequences on the x-axis and their known absolute copy numbers on the y-axis. Fourth, the total read count of all species in a sample is mapped onto the standard curve to calculate the total 16S rRNA gene copy number in the system, denoted as $n_{total}$, which is then converted to the total 16S rRNA gene copies using the formula:

$$N_{total} = n_{total}\times\frac{N}{10I}$$

Finally, the read counts of each taxonomic unit are mapped onto the standard curve to calculate the copy number $n_{x}$ for each unit, which is then converted to the absolute 16S rRNA gene copies for that taxon using:

$$N_{x} = n_{x} \times\frac{N}{10I}$$

This approach provides **absolute quantification of 16S rRNA genes** at multiple taxonomic levels.

**Copy number estimation with the ABI 3730 genetic analyzer**

The 16S rRNA gene amplicon sequence of bacteria in the sample are usually polymorphic in length and are close in length to the spike-in sequences (SPK). Before the final high-throughput sequencing is completed, it is impossible to obtain the proportion of SPK in the total sequence. When the proportion of SPK is inevitably too low or too high, it will cause a lot of waste of reagents and time. Therefore, we designed a method for predicting SPK copy number based on fluorescent capillary electrophoresis. The basic design is as follows: we design an indicator internal control whose copy number is the sum of the quantitative internal control copy number. At the same time, the length of its amplification product is about 100 bp longer than that of the amplification product of normal bacteria 16S rRNA gene, so that the two can be clearly distinguished. In fluorescence capillary electrophoresis, the peak area of the signal value output for each length of amplified product is proportional to the molar number of the amplified product. Therefore, the proportion of SPK can be estimated by the ratio of the target peak (containing the amplified product of the target bacterial 16S rRNA gene and quantitative SPK) to the indicator peak (the indicator internal control amplified product, whose molar number is equal to that of quantitative SPK).

The analysis process consists of several steps including: (1) The ZymoBIOMICS Microbial Community Standard (Zymo, D6305) was purchased, and its concentration was accurately determined using Qubit, then standardized to 10 ng/µl, designated as standard MSTD-1. MSTD-1 standard was mixed with hela cell genomic DNA at mass ratios of 1:9, 0.5:9.5, and 0.1:0.99 to prepare MSTD-2, MSTD-3, and MSTD-4, respectively. Additionally, three soil microbial DNA samples were collected and labeled MSTD-5, MSTD-6, and MSTD-7. (2) Take 10 ng MSTD (-1 to -7), add BSIS-4, BSIS-5, and BSIS-6, respectively, and perform PCR amplification using V4–V5 primers. After analyzing the amplification products by capillary electrophoresis on a 3730 gene analyzer, the proportion of BSIS in each reaction was estimated using PeakScan software (Figure S4 and Table S2). (3) All reactions were indexed by PCR to construct sequencing libraries, and 2 × 250 bp sequencing was performed using the Illumina Hiseq platform. The proportion of BSIS sequences in the sequencing data was analyzed (Table S2). Based on the above examples, we can conclude that the proportion of “quantitative internal reference” estimated based on the peak area ratio of capillary electrophoresis can accurately reflect the proportion of internal reference in the final sequencing library.

**An example for the calculation of the absolute quantitative copy number**

In order to speed up the subsequent operation, the spike reads were separated from the fastq to obtain microbe fastq and spike fastq. Then perform independent ASV analysis on the two groups of fastq after splitting. And the calculation of absolute quantitative copy number generally goes through three steps:

Firstly, according to the spike ASV abundance value and the absolute copy number data of spike added in the experiment, the linear regression method is used (first take the log10 of the data) to calculate the linear regression coefficient and obtain the relationship between addition and output. The specific process is as follows:

# ir_copies (Illumina run copies)

ir_copies = c(1.09e+6, 1.09e+6, 1.09e+5, 1.09e+5, 1.09e+4, 1.09e+4, 1.09e+3, 1.09e+3, 1.09e+3)

# Off-machine test reads

reads = c(23030, 30856, 2475, 3393, 303, 281, 36, 33, 20)
# ASV abundance

abundance = structure(c(2130, 19, 20, 7163, 4), names = c(‘ASV1’, ‘ASV2’, ‘ASV3’, ‘ASV4’, ‘ASV5’))

# Amount of template DNA on the machine

template_DNA_ng = 7.46 ng
# Library DNA amount

sample_DNA_ng = 2088.8 ng

# Sample weight

sample_quantity_g = 0.0348 g

# Log10 transformation
ir_copies_log10 = log10(ir_copies)
reads_log10 = log10(reads)
# Linear regression
lm_model = lm(reads_log10~ir_copies_log10)
lm_res = summary(lm_model)
# Coefficients
a = round(lm_res$coefficients[[2]], 4)
b = round(lm_res$coefficients[[1]], 4)
r2 = round(lm_res$r.squared, 4)

Secondly, the absolute copy number of microbe ASV was calculated based on the linear regression coefficient and the abundance value of microbe ASV. The specific process is as follows:

# Absolute copy number
absolute_copies = round(10^((log10(abundance) - b) / a))
# Absolute copy number at the DNA level (unit: copies/ng_dna)
dna_absolute = round(absolute_copies / template_DNA_ng)
# Sample level absolute copy number (unit: copies/g)
sample_absolute = round(absolute_copies / (sample_quantity_g * template_DNA_ng / sample_DNA_ng))

Finally, calculate the species copy number per unit DNA (copies/ng DNA) and the species copy number (copies/g (unit for solid or semi-solid objects like soil and feces), copies/mL (unit for liquid objects like water) sample, etc.) based on the amount of sequencing template DNA, sample DNA extraction volume, and sample volume used for DNA extraction. The specific process is as follows:

# 16S rRNA gene rdp-classifier copy number correction
copy = structure(c(3.92, 3.92, 3.92, 3.92, 4.68), names = c(‘ASV1’, ‘ASV2’, ‘ASV3’, ‘ASV4’, ‘ASV5’))

# Absolute copy number correction at the DNA level (unit: copies/ng_dna)
dna_absolute_correct = round(dna_absolute / copy)
# Sample level absolute copy number correction (unit: copies/g)
sample_absolute_correct = round(sample_absolute / copy)

**The accuracy validation of Accu16S/AccuITS**

To further illustrate the detection effectiveness and repeatability of the method, 24 soil samples from 8 sites (three replicates per site) were analyzed for absolute quantification of bacterial 16S rRNA genes. Approximately 500 mg of soil was used for each extraction. DNA was extracted using the FastDNA SPIN Kit for Soil (MP Biomedicals, 116560200), and the final DNA was eluted in 60 μL of elution buffer. DNA concentrations were accurately quantified using a Qubit fluorometer and normalized prior to downstream analysis. Each DNA sample was spiked with BSIS-6, BSIS-5, BSIS-4, and BSIS-3 sequences, followed by PCR amplification targeting the V4–V5 region of the 16S rRNA gene. The optimal spike-in ratio was determined using ABI 3730 sequencing. Libraries prepared with the optimal spike-in ratio were subjected to high-throughput sequencing. Based on the standard curve derived from the “quantitative reference sequences”, the total 16S rRNA gene copy number in each sample was calculated and converted to 16S rRNA gene copies per gram of soil, enabling absolute quantification of 16S rRNA genes across different taxonomic levels. To further validate the accuracy of Accu16S/AccuITS method, we compared the absolute quantification results using Accu16S with qPCR and ABI 3730 capillary electrophoresis methods for quantifying the absolute abundance of microorganisms in three ZYMO standards (ZYMO-GUT-1, ZYMO-GUT-2, ZYMO-GUT-3).

**More examples for the applications of Accu16S/AccuITS**

Using Accu16S absolute quantification, researchers studied the growth-promoting mechanism of potato rhizosphere bacterial communities [2]. The principal coordinate analysis found the rhizosphere soil community structure were significantly changed between continuous cropping (CC) and noncontinuous cropping (NCC) conditions on days 10 and 20 after emergence (Figure S5A). The CC soil at 20 days after emergence (DAE) (CC20) exhibited the highest absolute abundance of the top 10 bacterial phyla, notably Proteobacteria and Bacteroidetes (Figure S5B and Table S10). *Pantoea* sp. MCC16 was detected exclusively at 20 DAE in CC potatoes (Figure S5C), suggesting interactions between CC potato plants and *Pantoea* sp. MCC16. This study shows that absolute quantification reveals the recruitment of *Pantoea* sp. MCC16 by CC potatoes to alleviate continuous cropping obstacles.

Using Accu16S absolute quantification, researchers studied rhizosphere microbes of *Caragana korshinskii* with years of sand fixation [3]. N-fixing and phosphate-solubilizing genera like *Rhizobium*, *Ensifer*, and *Streptomyces* were significantly more abundant in 18-year samples, consistent in summer and fall (Figure S5D,E and Table S11). Redundancy analysis showed a strong positive correlation between dominant bacteria and available nitrogen (AN) (Figure S5F). Mantel tests revealed pH, total nitrogen, and N:P ratio significantly influenced bacterial communities across seasons (Figure S5G).

**REFERENCES**

1. Bai, Defeng, Chuang Ma, Jiani Xun, Hao Luo, Haifei Yang, Hujie Lyu, Zhihao Zhu*, et al.* 2025. “MicrobiomeStatPlots: Microbiome statistics plotting gallery for meta-omics and bioinformatics.” *iMeta* 4: e70002. <https://doi.org/10.1002/imt2.70002>

2. Ma, Haiyan, Zhitong Ren, Aihua Luo, Xiaoting Fang, Ruilin Liu, Chao Wu, Xinxin Shi*, et al.* 2025. “Self-alleviation of continuous-cropping obstacles in potato via root-exudate-driven recruitment of growth-promoting bacteria.” *Plant Communications* 6: 101372. <https://doi.org/10.1016/j.xplc.2025.101372>

3. Liu, Wangsuo, Kaiyang Qiu, Yingzhong Xie, Ruixia Wang, Haichao Li, Wenfen Meng, Yi Yang, Yeyun Huang, Yayuan Li, Yi He. 2022. “Years of sand fixation with Caragana korshinskii drive the enrichment of its rhizosphere functional microbes by accumulating soil N.” *PeerJ* 10: e14271. <https://doi.org/10.7717/peerj.14271>


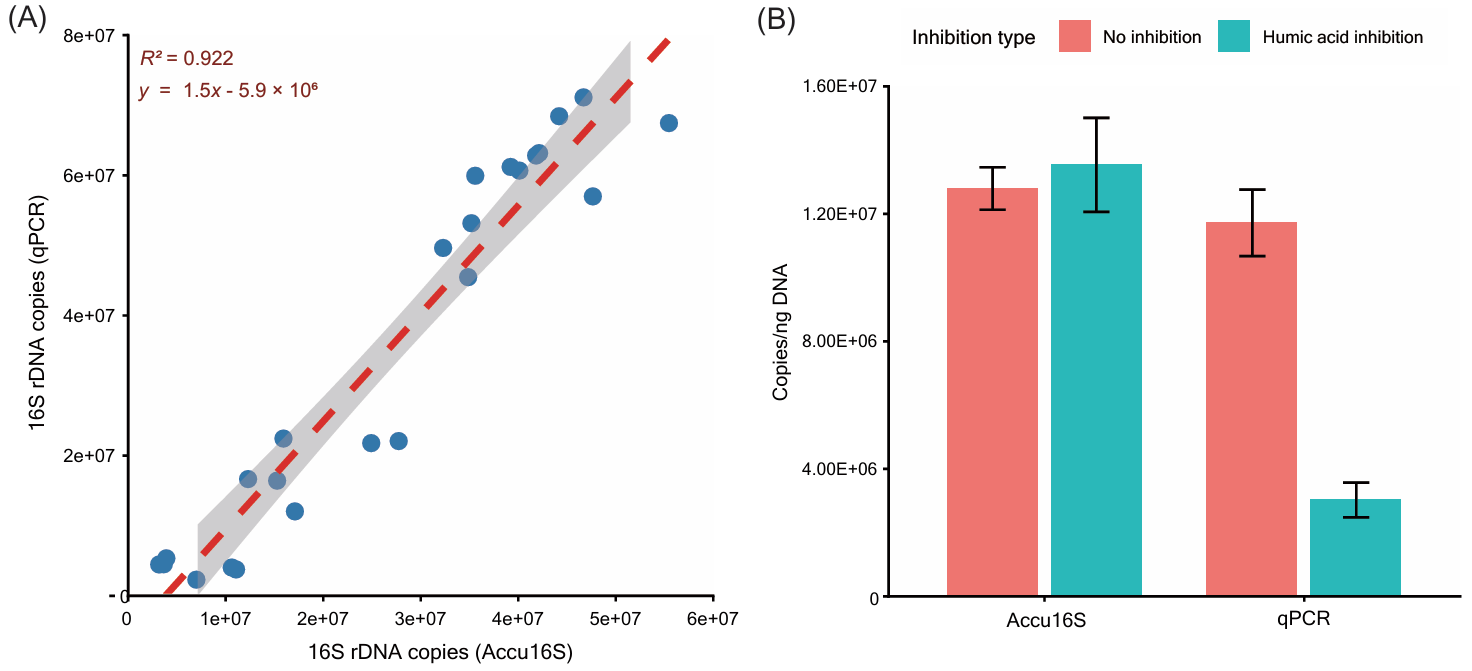
**Figure S1 Comparison between Accu16S/AccuITS and traditional quantitative Polymerase Chain Reaction (qPCR).** (A) Correlation between internal reference method (Accu16S/AccuITS) and qPCR method in detecting total 16S rRNA gene copy number. (B) The total 16S rRNA gene copy number was detected by qPCR and internal control method before and after adding humic acid inhibitor to soil DNA samples.


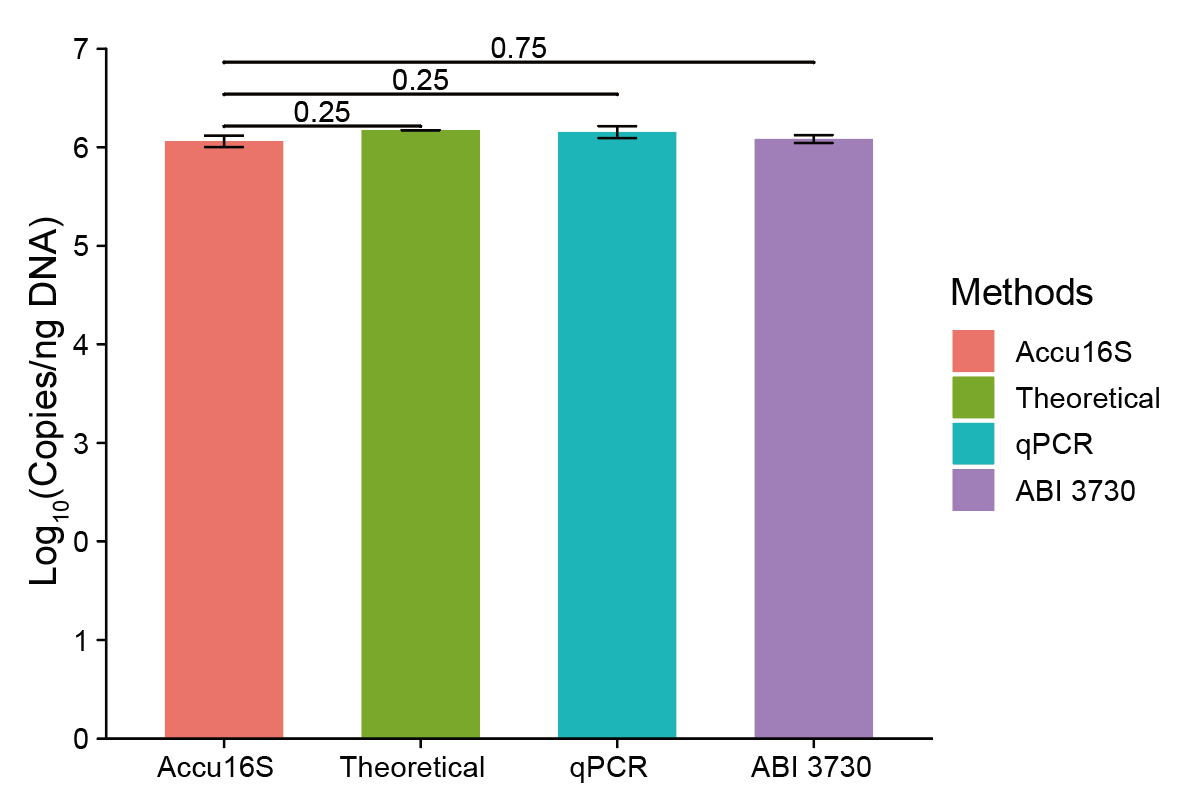


**Figure S2 Comparison of Accu16S method with other methods for absolute quantification of microorganisms.** The difference statistical test was performed on the absolute quantitative abundance and theoretical abundance of Accu16S in three ZymoBIOMICS Microbial Community (ZYMO) samples (ZYMO-GUT-1, ZYMO-GUT-2, ZYMO-GUT-3), as well as the results of quantitative Polymerase Chain Reaction (qPCR) and Applied Biosystems (ABI) 3730 capillary electrophoresis. The results showed that the absolute quantitative abundance of Accu16S was not significantly different from the theoretical abundance, qPCR, and ABI 3730 capillary electrophoresis results (*n* = 3 samples for each group, the significance test method used is two-sided Wilcoxon rank-sum test).


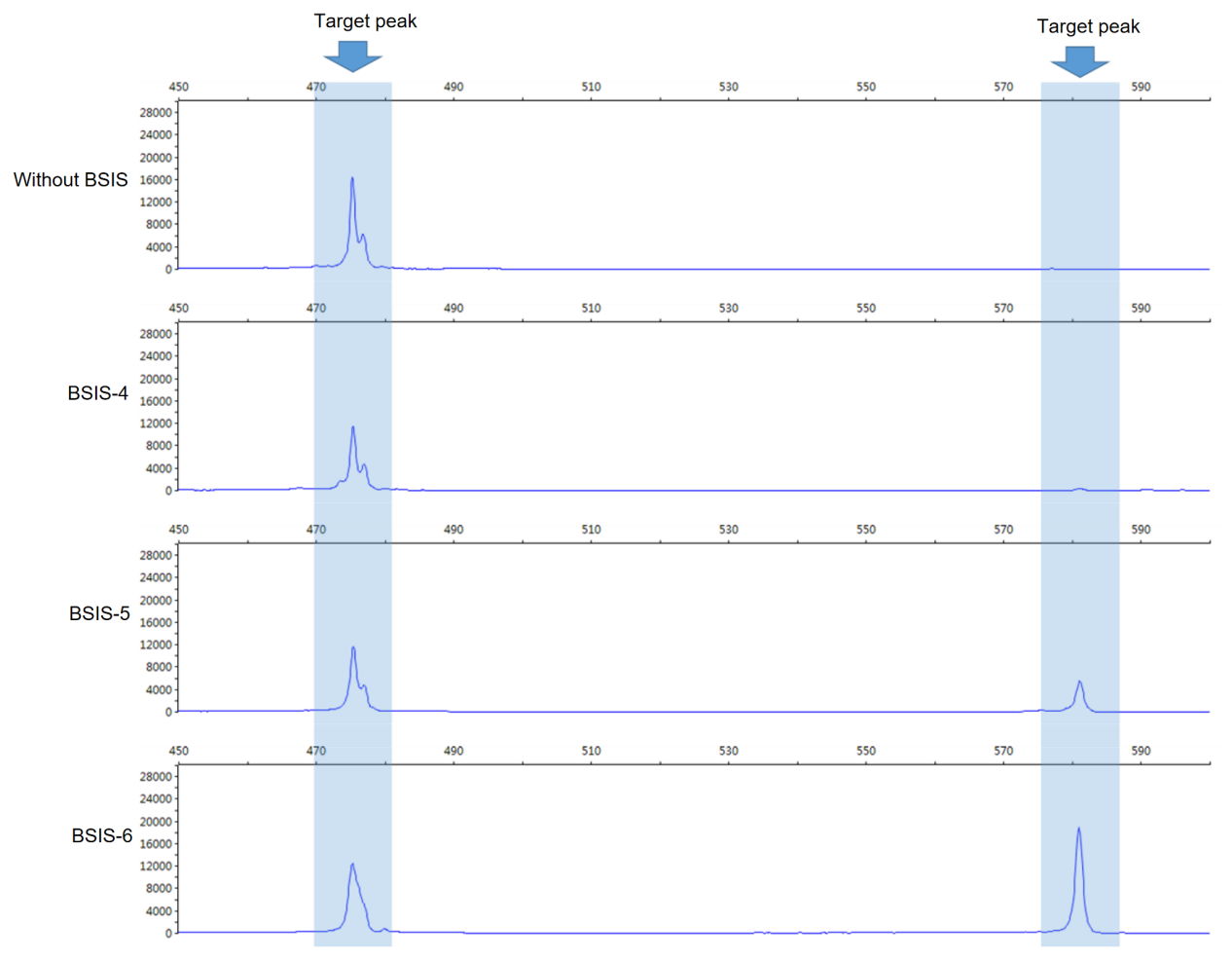


**Figure S3 The capillary electrophoresis results using PeakScan software.** Polymerase Chain Reaction (PCR) products are represented as peaks, with product length corresponding to the x-axis value of the peak and the absolute copy number corresponding to the peak area. The peak area can be calculated using PeakScan software. The target peak contains both sample DNA and the amplification product of the “quantitative internal control”, making them indistinguishable. The indicator peak only contains the amplification product of the “indicator internal control”. Since the ratio of the absolute copy numbers of the “indicator internal control” and the “quantitative internal control” is known (1:1 in this case), the peak area contributed by the “quantitative internal control” in the target band can be estimated from the peak area of the “indicator peak”. This value corresponds to the proportion of the “quantitative internal control” in the total sequence number. As shown in the figure, the indicator peak gradually becomes detectable with increasing Bacterial Spike-In Standards (BSIS) concentration, consistent with experimental expectations. Specific calculation results are shown in Table S2.


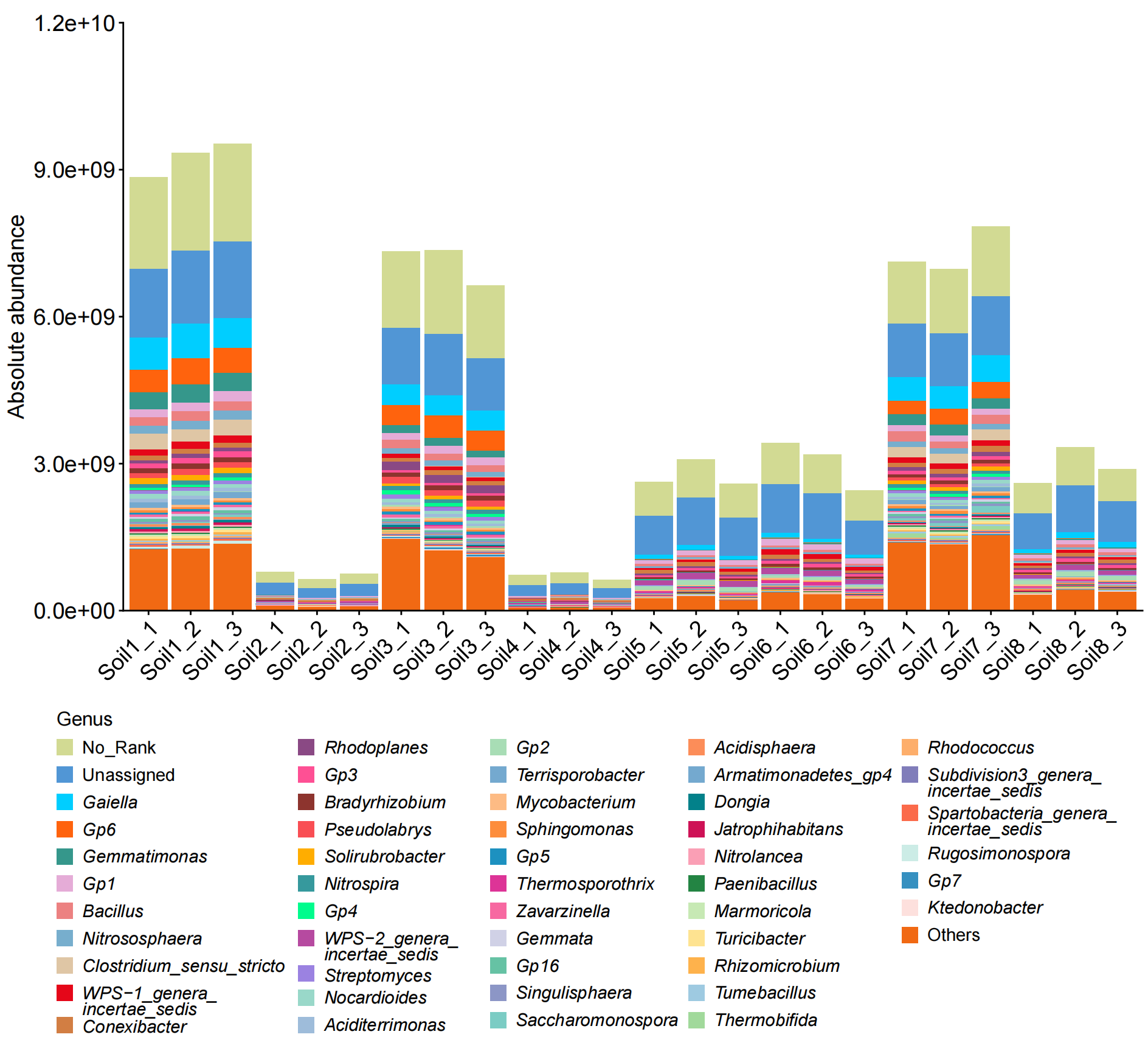


**Figure S4 The absolute copy number of 16S rRNA gene microorganisms at the level in real soil samples.** The absolute values of the 16S rRNA gene copy number of microorganisms in real soil samples detected using the method of this invention. “_1”, “_2”, and “_3” represent technical replicates of the same soil sample, indicating that the detection results of this method have high reproducibility.

**
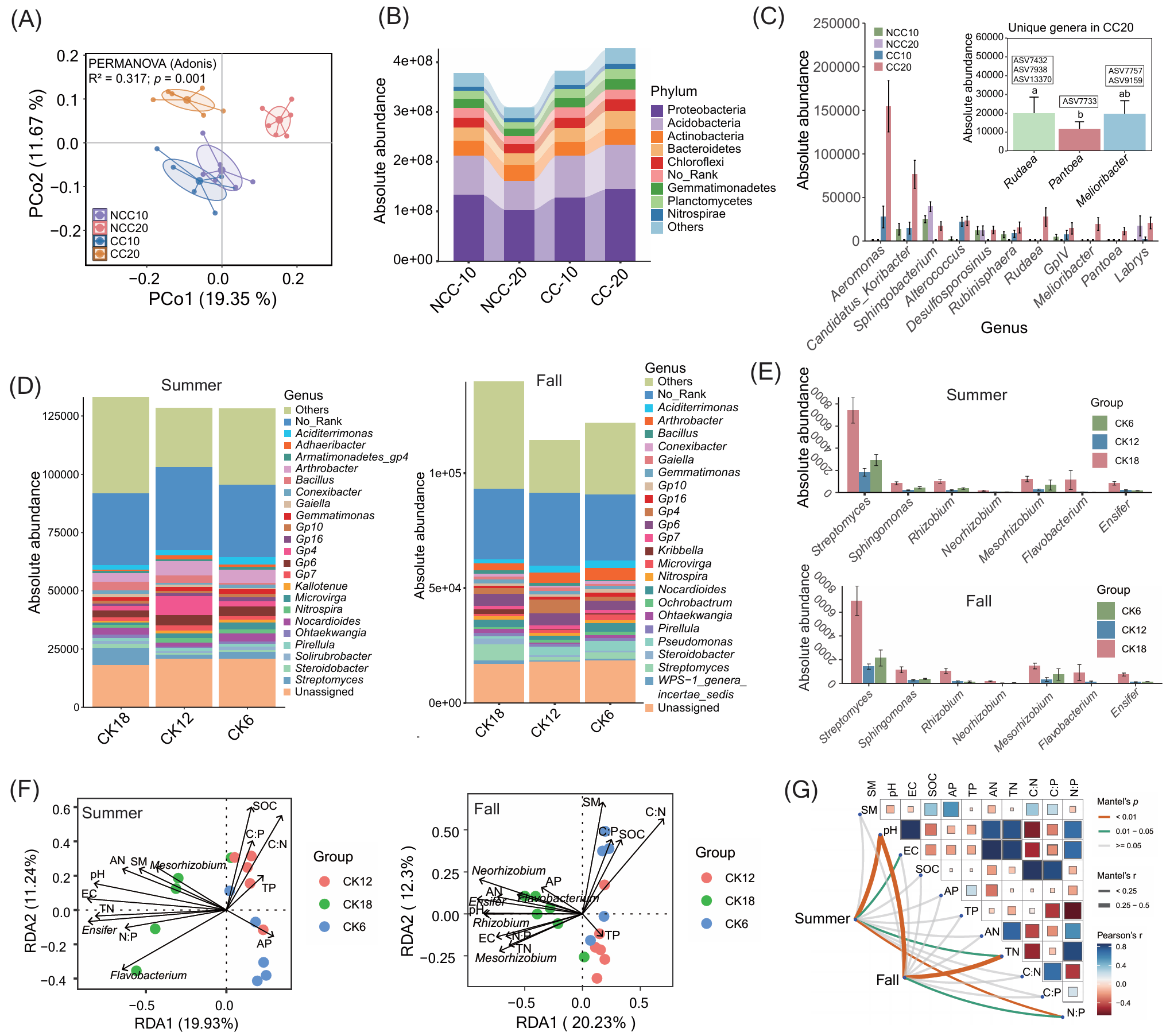
 Figure S5 Additional representative studies using Accu16S for microbial absolute quantification.** (A–C) The absolute abundance of bacteria significantly changed between continuous cropping (CC) and noncontinuous cropping conditions (NCC). (D–G) Accu16S analysis of *Caragana korshinskii* (CK) rhizosphere microbiota showed functional enrichment under long-term sand fixation, likely due to increased soil nitrogen. CK6, CK12, and CK18 indicate sand-fixing ages of 6, 12, and 18 years, respectively. SM, EC, SOC, AP, TP, AN, and TN indicate soil moisture, electrical conductivity, soil organic carbon, available phosphorus, total phosphorus, available nitrogen, and total nitrogen. C:N, C:P, and N:P denote the carbon-to-nitrogen, carbon-to-phosphorus, and nitrogen-to-phosphorus ratios.
